# Trend of Bronchial Hyperresponsiveness to Methacholine as a Cost Predictor of Mild-to-Moderate Asthma: A Twelve-Month Survey in Teenagers

**DOI:** 10.3390/children10121876

**Published:** 2023-11-29

**Authors:** Roberto W. Dal Negro, Paola Turco, Massimiliano Povero

**Affiliations:** 1 National Centre for Respiratory Pharmacoeconomics and Pharmacoepidemiology—CESFAR, 37124 Verona, Italy; robertodalnegro@cesfar.it (R.W.D.N.); turcop@libero.it (P.T.); 2 AdRes Health Economics and Outcomes Research, 10121 Turin, Italy

**Keywords:** mild-to-moderate asthma, teenagers, BHR as predictor of cost, long term outcomes, adherence to treatment

## Abstract

Bronchial asthma is characterized by variable airflow obstruction, airway inflammation, and bronchial hyperresponsiveness (BHR) to non-specific stimuli. The role of underlying airway inflammation and of related long-lasting BHR has been suboptimally investigated in teenagers with mild-to-moderate asthma, as has the corresponding economic impact over time. The aim of the present study was to calculate the cost of mild-to-moderate atopic asthma in teenagers arising from their degree of persisting BHR over a twelve-month period. Methods: Patients aged 12–18 years with mild-to-moderate symptoms treated with fluticasone fumarate/vilanterol 92/22 mcg daily were retrospectively followed for 12 months. Usual spirometric parameters, BHR to methacholine (MCh), and resource consumption (visits, hospitalizations, systemic steroids and/or antibiotics courses, school days off) were assessed at recruitment (the index date) and after 6 and 12 months. Adherence to treatment was also calculated. The cost of asthma was calculated based on Italian tariffs and published papers. The trend over time in BHR and the association between response to MCh and total cost were investigated by using regression models adjusted for repeated measures. Results: 106 teenagers (53 males, age 15.9 ± 1.6 years) were investigated. The annual cost of asthma proved significantly related to the BHR trend: every increment of a factor 10 in the response to MCh was associated with a saving of EUR 184.90 (95% CI −305.89 to −63.90). BHR was progressively optimized after 6 and 12 months in relation to the patients’ compliance to treatment (≥70% of prescribed inhalation doses). Conclusions: the usual spirometric parameters are largely insufficient to reflect the effects of underlying persistent inflammation in milder forms of asthma in teenagers. In terms of clinical governance, the periodic assessment of non-specific BHR is the appropriate procedure from this point of view. Non-specific BHR proves a reliable procedure for predicting and monitoring the economic impact of mild-to-moderate asthma in teenagers over time.

## 1. Introduction

Bronchial asthma is a disease condition characterized by variable airflow obstruction, airway inflammation, and bronchial hyperresponsiveness (BHR) to non-specific stimuli that cause respiratory symptoms such as coughing, wheezing, and attacks of breathlessness [1,2,3,4,5]. 

Checking of asthma control is usually pursued by a combination of different tools, such as periodic reports of symptoms (such as by specific questionnaires such as the Asthma Control Test–ACT [6,7] or Childhood Asthma Control Test [8], the incidence of exacerbations, the count of rescue drugs used in a fixed period (i.e., daily, weekly, monthly); measures of markers of type-2 inflammation in the airways (namely, fractional exhaled nitric oxide—FeNO [9], and lung function tests (currently, peak flow measurements at home or laboratory forced expiratory volume in 1 s [FEV_1_]) [10,11,12,13,14]. 

Unfortunately, as the widely used spirometric parameters measured at rest (i.e., FEV_1_ or forced vital capacity) are frequently inconclusive and result in the normal range in mild-to-moderate atopic asthma, they cannot inform about the effective role of underlying inflammation of the airways that is the main basic condition sustaining persistent asthma [15,16,17,18,19]. 

In contrast, non-specific BHR is a peculiar condition that is strictly related to the presence of variable degrees of active airway inflammation in atopic asthma [2,13]. Indeed, the periodic assessment of non-specific BHR (namely the response to increasing doses of methacholine [MCh]) has also long been recommended in young asthmatics for assessing and monitoring the underlying airway inflammation as a cause of sustained asthma variability [20]. Indeed, MCh is a non-specific cholinergic agonist (parasympathomimetic agent) that acts through muscarinic receptors in the lungs to induce bronchoconstriction, and the bronchoprovocation test with MCh is primarily used to diagnose and grade bronchial hyperreactivity, which is the hallmark of asthma.

Several conditions can affect BHR, namely, exposure to allergens and/or irritants, smoke, environmental factors, weather changes, dry air, hypo-osmolar agents, and physical exercise. It should be taken into account that the extent of non-specific BHR and corresponding asthma variability can also be primarily related to patients’ behaviour. In fact, a very common cause that is able to substantially affect BHR is the patients’ compliance with treatment, particularly in children and teenagers [21]. Suboptimal or insufficient compliance with inhalation therapy is a condition well known to cause the persistency of airway inflammation and consequently of BHR, and to increase the risk of uncontrolled asthma [22,23], thus leading to higher cost of illness particularly in young patients [24].

The aim of the present study was to investigate the clinical outcomes and calculate the cost of treating mild-to-moderate atopic asthma in teenagers according to the extent of persisting BHR over a twelve-month period.

## 2. Materials and Methods

The study was an observational, retrospective analysis of asthmatic teenagers referring to the Lung Unit of the Specialist Medical Centre (CEMS), Verona, Italy, over the period February 2018–September 2019. Data were obtained from the institutional UNI EN ISO 9001–2008 validated database, and classic Boolean algebraic formulas were used for subject selection [25]. The database contains historical (namely, personal and familial), clinical, and health economic data, complete lung function, and continuously updated therapeutic information for each patient referring to the Centre. The present study is a post hoc analysis stemming from a previous survey that investigated the effect of non-compliance to inhalation therapy on outcomes in teenagers with mild-to-moderate atopic asthma [24].

Selection criteria were: non-smoker teenagers (ranging 12–18 years of age) of both genders with mild-to-moderate atopic asthma and without any relevant comorbidity who had been prescribed fluticasone fumarate/vilanterol 92/22 mcg dry powder o.d. via Ellipta. This combination was chosen because it is allowed in the range of 12–18 years of age in our country. Exclusion criteria were: the presence of physical or cognitive limitations affecting inhalation procedures; lack of parents’ informed consent; incomplete clinical and/or lung function data; prescriptions of ICS/LABA o.d. different from fluticasone fumarate/vilanterol via Ellipta. 

In addition to sex, age, and FEV_1_, maximum mid-expiratory flow (MMEF) and maximum expiratory flow at 25% of lung filling (MEF_25_) were assessed at recruitment (the index date) and after 6 and 12 months. Lung function was assessed by means of the MiniBox^TM^ (PulmOne Advanced Medical Devices, Ltd., Ra’anana, Israel), and data were reported as % predicted. At the same time, the bronchial response to MCh was also assessed in all subjects and reported as the MCh dose (in mcg) inducing a 20% drop from their FEV_1_ baseline value (PD_20_ FEV_1_). Clinical outcomes collected over the study period included the number of hospitalizations, the duration of each hospital stay (in days), the number of exacerbations, the number of school or work days off, the number of general practitioner (GP) or specialist visits, the numbers of systemic steroids and of antibiotics courses prescribed. At recruitment (the index date), these health outcomes were calculated over the previous six months and compared with those recorded after 6 and 12 months of the study. 

As compliance with inhalation treatments represents a critical aspect of asthma management, particularly in young patients [26], the compliance with treatment was also calculated in this study. Given that the inhaler device used (Ellipta) is provided with a clearly visible dose counter and contains doses of drugs enough for thirty days of treatment, the degree of adolescents’ adherence to the inhalation regimen was checked every month via telephone calls. Each adolescent (or one of the adolescents’ parents) had then to communicate the number of remaining doses visible in his/her device to the interviewer, and the compliance was calculated as the percentage of skipped inhalation doses per month, which corresponded to days of treatment skipped over each period of the survey. As previously consolidated, subjects were defined as “non-compliant” or “compliant” if they assumed <70% or ≥70% of prescribed inhalation doses, respectively [26].

The study was approved by the R&CG Ethical Committee during the session officially held on 10 October (code: 02/RG02/2017). Though the selection of subjects was conducted by automatic procedures from the database, informed consent from teenagers’ parents was requested.

### 2.1. Economic Evaluation

Total cost in each period was calculated according to the societal perspective, including both direct healthcare costs (visits, hospitalizations, and drugs) and indirect costs (parents’ loss of productivity due to school days off).

The cost of antibiotics was evaluated by considering the cost of 3 packs of cefuroxime (500 mg, 6 tablets) for each cycle. The cost of systemic steroids was evaluated by considering the cost of 1 pack of prednisone (25 mg, 10 tablets) for each cycle. For both antibiotics and systemic steroids, the reference prices for each product package were used [27] corresponding to EUR 6.16 for cefuroxime and EUR 5.87 for prednisone. 

For hospitalizations, the tariff of the Diagnosis Related Group (DRG) 98, “Bronchitis and asthma, age < 18 years”, was used [28]. The cost was calculated as the mean between the tariffs for hospital admission longer than 1 day (EUR 1538) and day hospital (EUR 185) weighted for the number of hospital discharges reported in Italy (90% ordinary hospitalization and 10% day hospital) [29]. The duration was not considered in the analysis as the DRG tariff covers all the length of the hospital stay. The cost of each visit was equal to EUR 20.66 according to the national tariff [28].

The indirect costs were estimated by multiplying the number of school days lost due to the disease by the average daily salary (EUR 82.15) in Italy [30] updated to 2022 [31]. The lowest available value was considered, conservatively.

### 2.2. Statistics

Categorical variables were expressed as counts and percentages, continuous variables were summarized using the mean and standard deviation (SD). Continuous baseline characteristics (i.e., age, FEV_1_, MMEF, MEF_25_, and PD_20_ FEV_1_) were compared between compliant and non-compliant patients by using a non-parametric Wilcoxon Mann Whitney test. Gender-related analysis was conducting using a two-sided exact Fisher test. 

Differences in response to MCh between pre- and after 6 and 12 months post-treatment were tested using a generalized estimating equation (GEE) model [32], with a covariate of treatment period (gamma family, identity as link function). The effects of MCh or time on the outcomes were expressed in terms of mean difference and 95% confidence intervals (CI). To take into account the potential interaction between time and compliance, the interaction term “compliance x time” was added to the models, representing the effect of compliance during the study period (i.e., after 6 and 12 months of treatment). 

The evolution both of clinical outcomes (number of exacerbations, courses of antibiotics and systemic steroids, GP and specialist visits, hospitalization, and number of school or work days off) and total cost (detailed in drugs, visits/hospitalization, and indirect cost due to absenteeism) was estimated with a series of GEE models with a covariate of treatment period (after 6 and 12 months).

The associations between response to MCh and both clinical outcomes and total cost were investigated with the same approach, considering the log10-transformed PD_20_ FEV_1_ (due to the expected skewness of the variable PD_20_ FEV_1_) as an independent predictor. As explorative analysis, response to MCh was also investigated as a categorical predictor based on the thresholds PD_20_ FEV_1_ < 400 mcg (high responders), PD_20_ FEV_1_ 400–800 mcg (medium responders), and PD_20_ FEV_1_ > 800 mcg (low responders). 

All models were adjusted by age and gender by including the two variables as covariates in the GEE models. Statistical analysis was performed using STATA (StataCorp. 2017. Stata Statistical Software: Release 15. College Station, TX, USA: StataCorp LLC).

## 3. Results

The whole sample consisted of 106 teenagers (60.4% males). The mean age at recruitment was 15.9 years (SD = 1.6) (Table 1). At baseline, spirometric indices showed the presence of mild airway obstruction, mainly involving peripheral airways.

According to the protocol, 53 patients were classified as compliant and 53 as non-compliant (Table 1) with inhalation treatment. Demographic and lung function parameters at recruitment were similar in the two subgroups (based on a *p*-value threshold of 0.05) with the exception of gender, as the prevalence of males was higher in the non-compliant group.

At baseline, spirometric indices showed a lung function pattern characterized by mild airway obstruction, mainly involving peripheral airways. As the basal impact seems mild, it might be assumed that all subjects were presumably already assuming ICS/LABA, though to a variable degree of adherence.

**Figure 1:**
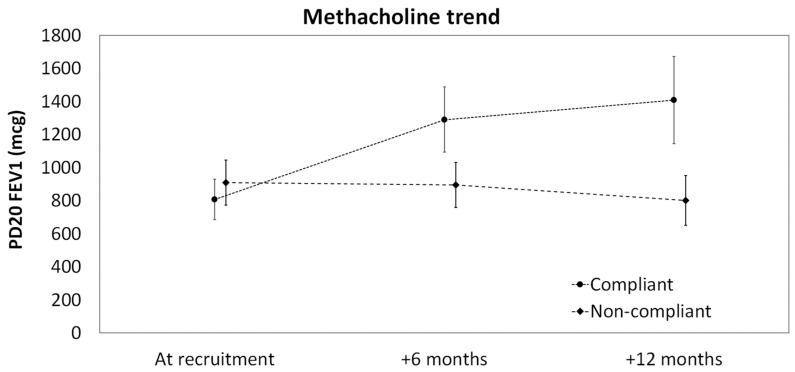
Trend of methacholine from recruitment up to 12 months post-treatment for compliant and non-compliant patients (dots and diamonds represent mean values, bars represent 95% confidence intervals).

The trend of PD_20_ FEV_1_ is reported in Figure 1 and was confirmed by the GEE model (Table 2). At baseline, PD_20_ FEV_1_ was slightly lower among compliant patients than non-compliant patients (−107.9, 95% CI −308.3 to 92.5). Over time, no significant changes from baseline were observed in the non-compliant group after both 6 months (−7.31 mcg, 95% CI −168.8 to 154.2) and 12 months (−97.6 mcg, 95% CI −286.2 to 91.1) of treatment, while in the compliant group the mean PD_20_ FEV_1_ increased of +493.8 mcg (95% CI 260.8 to 726.9) and +710.2 mcg (95% CI 453.8 to 966.7) after 6 and 12 months, respectively (Table 2).

Almost all clinical outcomes decreased from baseline up to 12 months in compliant patients (Table 3). The reduction was more pronounced during the first 6-month period. Resource consumption remained stable in non-compliant patients. The number of hospitalizations was negligible during the first months of observations (0.05 at recruitment and 0.07 after 6 months); no hospitalizations were recorded after this period.

The mean total cost per patient was EUR 364.15 (SD = 385.81) at recruitment and in the subgroup of complaint patients progressively decreased over the 12 months of the survey (Table 4) as a direct consequence of the reduction observed in almost all clinical outcomes. The reduction was more pronounced in the second (EUR −386.03, 95% CI −520 to −252) than in the first post-treatment semester (EUR −276.96, 95% CI −443 to −111). In non-compliant patients, total cost increased in the first semester (mainly due to visits and hospitalizations).

Response to MCh seemed to affect all the clinical outcomes, considering PD_20_ FEV_1_ as both continuous (Table 5) and categorical (Table 6). Considering PD_20_ FEV_1_ as a continuous variable, every increment of a factor of 10 in PD_20_ FEV_1_ (e.g., if response to MCh increased from 10 to 100 mcg or from 400 to 4000 mcg) was associated with a reduction in the numbers of exacerbations (−0.36, 95% CI −0.66 to −0.07), antibiotics courses (−0.31, 95% CI −0.49 to −0.12), systemic steroids courses (−0.24, 95% CI −0.46 to −0.01), GP visits (−0.73, 95% CI −1.23 to −0.23), specialist visits (−0.05, 95% CI, −0.08 to −0.02), hospitalizations (−0.05, 95% CI −0.08 to −0.02), and school/work days off (−1.02, 95% CI −1.91 to −0.12). A similar trend was observed considering the response to methacholine as a categorical variable according to pre-specified thresholds (Table 6). 

Response to MCh seemed also to affect the total cost, considering PD_20_ FEV_1_ as both continuous and categorical (Table 7). Considering PD_20_ FEV_1_ as a continuous variable, every increment of a factor of 10 in PD_20_ FEV_1_ (e.g., if response to MCh increased from 10 to 100 mcg or from 400 to 4000 mcg) was associated with savings of EUR 184.90 (95% CI −305.89 to −63.90). According to pre-specified thresholds, the mean cost of patients with medium response to MCh (PD_20_ FEV_1_ 400–800 mcg) and those with low response (PD_20_ FEV_1_ > 800 mcg) was lower than the cost of patients with high response: EUR −141.67 (95% CI −305.74 to 22.40) and EUR −208.55 (95% CI −358.48 to −58.61), respectively.

## 4. Discussion

Several longstanding studies have underlined that the early recognition of bronchial asthma is essential for achieving effective control and/or preventing the progressive worsening of asthma [33,34,35,36]. Nevertheless, bronchial asthma of mild severity [1] is still currently largely underestimated, underinvestigated, or neglected, particularly in teenagers. Approaches are mainly based on addressing symptoms reported by patients or their relatives, but the careful investigation of asthma by means of appropriate lung function tests is still insufficient in these cases. On the other hand, usual spirometric indices are frequently inconclusive in mild-to-moderate atopic asthma as they do not give reliable information concerning the underlying airway inflammation [15,16,17,18,19,20]. For these reasons, BHR to MCh was chosen in the present study as the most suitable indicator.

Unfortunately, though recommended [37], the periodic assessment of non-specific BHR aiming to check the degree of variability in inflammation-induced asthma and the effects of preventive drugs is still scarcely used in our country, even by lung physicians operating in specialist institutional settings (around in 8–9% of cases).

Data from the present study prove that substantial differences emerge over time in terms of annual cost of asthma among teenagers characterized by different degrees of non-specific BHR. In other words, subjects with sustained milder responsiveness to MCh were those characterized by a much lower consumption of health resources and annual cost coupled with lower morbidity and higher asthma control at six and twelve months. Note that these differences also appear strictly related to the teenagers’ compliance with the regular use of inhaled preventive drugs in our study. These results clearly confirm the high informative power of non-specific BHR concerning the role of uncontrolled inflammation in underlying asthma variability. Similar results were obtained in other studies that investigated the role of BHR from this point of view, though the related economic impact was not valued [22,23,24,38,39].

To the best of our knowledge, the present survey is the first to have used the periodic assessment of non-specific BHR as a predictor of mild-to-moderate asthma cost in teenagers over twelve months. Actually, direct and indirect costs proved significantly and directly related to the severity of non-specific BHR over twelve months. These results clearly support the evidence that non-specific BHR can predict the economic effect of suboptimal control of airway inflammation underlying mild-to-moderate asthma regardless of its specific causes. However, as sub-optimal compliance with the regular administration of inhaled drugs represents the most frequent cause of asthma variability and poor asthma control in teenagers due to variable cultural, educational, behavioral, and psychological conditions that can frequently limit asthma management [19,21,40,41,42,43,44,45], compliance with treatment should always be carefully investigated before modifying the current therapeutic strategy. 

Some limitations of the present study are: (a) the survey is monocentric and the sample size is limited; (b) drugs to inhale were only prescribed via DPIs in order to favor the periodic calculation of teenagers’ compliance at home; (c) due to the age-dependent prescription of different ICS and LABA molecules in our country, once- and twice-daily inhalation regimens were pooled in the present study. Points of strength are: (a) the sample of subjects was automatically obtained from the central database by means of pre-defined Boolean equations strictly corresponding to the inclusion and exclusion criteria; (b) though the cost of asthma is generally related to the degree of severity [46], the cost of mild-to-moderate atopic asthma in teenagers was for the first time assessed by means of the trend of non-specific BHR to MCh over twelve months because it is much more relevant to persistent active airway inflammation than the usual spirometric indices; (c) costs were also related to subjects’ compliance with their daily therapeutic strategy.

## 5. Conclusions

Some messages seem to emerge from the present study. Mild-to-moderate asthma should not be underestimated in teenagers as they usually tend to minimize symptoms and asthma impact due to cultural, educational, and psychological factors. Careful attention is required from the diagnostic point of view in these cases as usual spirometric indices can frequently be inconclusive in milder forms of asthma because they are largely insufficient to inform about the underlying persistent airway inflammation. Asthma can be effectively investigated and monitored in these young patients by means of more specific lung function tests, namely by the periodic assessment of non-specific BHR, which is the appropriate procedure from this point of view. Furthermore, the assessment of non-specific BHR can be also used as a reliable tool for calculating and monitoring the economic impact of mild-to-moderate asthma in teenagers over time. Finally, the data of the present investigation seem to show that the periodic assessment of BHR can also be helpful in checking indirectly the adherence of teenagers to their therapeutic strategy even if this is based on the once-daily administration of long-acting inhalation drugs, which is presumed to highly support the patients’ compliance over time. 

## Figures and Tables

**Table 1:** Demographic and lung function parameters at recruitment.

Variables	Total (*n* = 106)	Compliant (*n* = 53)	Non-Compliant (*n* = 53)	*p*-Value
% male	60.4%	54.7%	66.0%	0.160
Mean age (SD)	15.9 (1.6)	15.5 (1.7)	16.2 (1.5)	0.050
Mean FEV_1_ (SD)	85.8 (14.7)	85.2 (15.5)	87.4 (14.1)	0.532
Mean MMEF (SD)	52.8 (18.7)	51.4 (17.3)	52.9 (19.4)	0.745
Mean MEF_25_ (SD)	45.2 (19.1)	44.8 (18.1)	45.1 (19.5)	0.953
Mean PD_20_ FEV_1_ (SD)	857.6 (482.9)	807.1 (411.0)	908.1 (544.7)	0.358

FEV_1_: forced expiratory volume in 1 s; MMEF: maximum mid-expiratory flow; MEF_25_: maximum expiratory flow at 25% of lung filling; PD_20_ FEV_1_: MCh dose (in mcg) inducing a 20% drop from the FEV_1_ baseline value; SD: standard deviation.

**Table 2:** Effect of time and compliance in the evolution of PD_20_ FEV_1_ values.

Predictors	Estimate (95% CI)
Compliant vs. non-compliant at baseline	−107.9 (−308.3 to 92.5)
Time effect (vs. at recruitment) in non-compliant patients	
+6 months post-treatment	−7.31 (−168.8 to 154.2)
+12 months post-treatment	−97.6 (−286.2 to 91.1)
Interaction: time effect (vs. at recruitment) in compliant patients	
+6 months post-treatment	493.8 (260.8 to 726.9)
+12 months post-treatment	710.2 (453.8 to 966.7)

CI: confidence interval.

**Table 3:** Clinical outcomes at baseline (mean and standard deviation) and clinical evolution over 12 months (mean and 95% confidence interval) in compliant and non-compliant patients.

Clinical Outcomes	At Recruitment	Δ 6 Months vs. at Recruitment		Δ 12 Months vs. at Recruitment	
		Compliant	Non-Compliant	Compliant	Non-Compliant
Exacerbation rate	0.87 (0.85)	−0.47 (−0.76 to −0.18)	0 (−0.29 to 0.29)	−0.66 (−0.92 to −0.40)	0.13 (−0.19 to 0.46)
Antibiotics courses	0.89 (0.87)	−0.53 (−0.78 to −0.27)	0.04 (−0.24 to 0.31)	−0.75 (−0.99 to −0.52)	−0.11 (−0.42 to 0.19)
Systemic steroids courses	0.84 (0.76)	−0.66 (−0.88 to −0.44)	−0.09 (−0.35 to 0.16)	−0.79 (−1.01 to −0.57)	−0.08 (−0.41 to 0.26)
GP visits	1.44 (1.06)	−0.91 (−1.19 to −0.62)	0.23 (−0.10 to 0.55)	−1.25 (−1.51 to −0.98)	0.11 (−0.22 to 0.45)
Specialist visits	1.58 (1.18)	−0.72 (−1.06 to −0.38)	0.11 (−0.19 to 0.42)	−0.81 (−1.15 to −0.48)	0.09 (−0.32 to 0.50)
Hospitalizations	0.05 (0.21)	−0.04 (−0.14 to 0.07)	0.08 (0.004 to 0.15)	-	-
Number of days off	2.60 (2.18)	−2.15 (−2.69 to −1.61)	0.23 (−0.27 to 0.72)	−2.34 (−2.94 to −1.74)	0.02 (−0.67 to 0.71)

GP: general practitioner; Δ: delta (mean difference).

**Table 4:** Cost at baseline (mean and standard deviation) and changes over 12 months (mean and 95% confidence interval) in compliant and non-compliant patients.

Cost Item (EUR)	At Recruitment	Δ 6 Months vs. at Recruitment		Δ 12 Months vs. at Recruitment	
		Compliant	Non-Compliant	Compliant	Non-Compliant
Drugs	21.32 (17.75)	−13.64 (−18.9 to −8.4)	0.14 (−5.1 to 5.3)	−18.60 (−23.4 to −13.8)	−2.54 (−8.5 to 3.4)
Visits and hospitalization	128.94 (298.47)	−86.62 (−236 to 62.3)	113.21 (11.9 to 215)	−175.2 (−287 to −63.9)	4.29 (−6.4 to 15.0)
Indirect	213.9 (178.79)	−176.70 (−221 to −132)	18.6 (−22.0 to 59.2)	−192.20 (−241 to −143)	1.55 (−55.1 to 58.2)
Total	364.15 (385.81)	−276.96 (−443 to −111)	131.95 (18.0 to 246)	−386.03 (−520 to −252)	3.30 (−55.7 to 62.3)

Δ: delta (mean difference).

**Table 5:** Results of GEE analysis of the association between response to methacholine and clinical outcomes over 12 months.

Clinical Outcome	Estimate (95% CI)
Exacerbation rate	−0.36 (−0.66 to −0.07)
Antibiotics courses	−0.31 (−0.49 to −0.12)
Systemic steroids courses	−0.24 (−0.46 to −0.01)
GP visits	−0.73 (−1.23 to −0.23)
Specialist visits	−0.15 (−0.39 to 0.10)
Hospitalizations	−0.05 (−0.08 to −0.02)
Number of days off	−1.02 (−1.91 to −0.12)

GP: general practitioner; CI: confidence interval.

**Table 6:** Results of GEE (scenario) analysis of the association between response to methacholine (considered as categorical variable) and clinical outcomes over 12 months.

Clinical Outcome	PD_20_ FEV_1_ Strata	Estimate (95% CI)
Exacerbation rate	400–800 vs. <400 mcg	−0.43 (−0.78 to −0.09)
	>800 vs. <400 mcg	−0.53 (−0.81 to −0.24)
Antibiotics courses	400–800 vs. <400 mcg	−0.19 (−0.51 to 0.13)
	>800 vs. <400 mcg	−0.34 (−0.58 to −0.10)
Systemic steroids courses	400–800 vs. <400 mcg	−0.29 (−0.66 to 0.08)
	>800 vs. <400 mcg	−0.24 (−0.53 to 0.06)
GP visits	400–800 vs. <400 mcg	−0.50 (−0.97 to −0.02)
	>800 vs. <400 mcg	−0.68 (−1.09 to −0.26)
Specialist visits	400–800 vs. <400 mcg	0.00 (−0.47 to 0.47)
	>800 vs. <400 mcg	−0.20 (−0.53 to 0.13)
Hospitalizations	400–800 vs. <400 mcg	−0.07 (−0.16 to 0.02)
	>800 vs. <400 mcg	−0.06 (−0.14 to 0.02)
Number of days off	400–800 vs. <400 mcg	−0.25 (−1.14 to 0.64)
	>800 vs. <400 mcg	−1.02 (−1.72 to −0.32))

GP: general practitioner; PD_20_ FEV_1_: MCh dose (in mcg) inducing a 20% drop from their FEV_1_ baseline value.

**Table 7:** Results of GEE analysis of the association between response to methacholine and total cost over 12 months.

**Model 1**	**Estimate (95% CI)**
Log_10_ PD_20_ FEV_1_	−184.90 (−305.89 to −63.90)
**Model 2**	**Estimate (95% CI)**
PD_20_ FEV_1_ 400–800 vs. <400 mcg	−141.67 (−305.74 to 22.40)
PD_20_ FEV_1_ > 800 vs. <400 mcg	−208.55 (−658.48 to −58.61)

PD_20_ FEV_1_: MCh dose (in mcg) inducing a 20% drop from the FEV_1_ baseline value; CI: confidence interval.

## Data Availability

The data presented in this study are available on request from the corresponding author. The data are not publicly available due to privacy.
